# Exploring mechanisms and contexts in a Peer Education Project to improve mental health literacy in schools in England: a qualitative realist evaluation

**DOI:** 10.1093/her/cyad026

**Published:** 2023-07-24

**Authors:** E L Curtin, E Widnall, S Dodd, M Limmer, R Simmonds, A E Russell, A Kaley, J Kidger

**Affiliations:** Department of Population Health, London School of Hygiene and Tropical Medicine, Keppel Street, London WC1E 7HT, UK; Population Health Sciences, Bristol Medical School, University of Bristol, Canynge Hall, Bristol BS8 2PS, UK; Division of Health Research, Lancaster University, Hazelrigg Lane, Lancaster LA1 4YW, UK; Division of Health Research, Lancaster University, Hazelrigg Lane, Lancaster LA1 4YW, UK; Mental Health Foundation, Long Lane, London SE1 4PD, UK; College of Medicine and Health, University of Exeter, Heavitree Road, Exeter EX1 2LU, UK; Division of Health Research, Lancaster University, Hazelrigg Lane, Lancaster LA1 4YW, UK; School of Health and Social Care, University of Essex, Wivenhoe Park, Colchester CO4 3SQ, UK; Population Health Sciences, Bristol Medical School, University of Bristol, Canynge Hall, Bristol BS8 2PS, UK

## Abstract

Poor adolescent mental health calls for universal prevention. The Mental Health Foundation’s ‘Peer Education Project’ equips older students (‘peer educators’) to teach younger students (‘peer learners’) about mental health. The peer-led lessons cover defining good and bad mental health, risk and protective factors, self-care, help-seeking and looking after one another. While previous pre-post evaluations have suggested effectiveness, the mechanisms through which the intervention improves mental health literacy remain unclear. We purposively recruited seven secondary schools across England from 2020 to 2022 and collected data through five observations, 12 staff interviews and 15 student focus groups (totalling 134 students; 46 peer educators aged 14–18 years and 88 peer learners aged 11–13 years). Our realist analysis adopted retroductive logic, intertwining deductive and inductive approaches to test the initial programme theory against insights arising from the data. We developed Context–Mechanisms–Outcome configurations related to four themes: (i) modelling behaviours and forming supportive relationships, (ii) relevant and appropriate content, (iii) peer educators feeling empowered and (iV) a school culture that prioritises mental health support. Our refined programme theory highlights key mechanisms, contexts conducive to achieving the outcomes and ways to improve training, recruitment and delivery to maximise effectiveness for similar peer-led initiatives.

## Introduction

Globally, an estimated half of all lifetime mental disorders commence before the age of 14 years [1]. In England, one in six children aged 7–16 years and one in four 17- to 19-year-olds suffer from a diagnosable mental health condition, which worsened considerably over the coronavirus disease 2019 (COVID-19) pandemic [2]. Mental health literacy (MHL) is defined as an understanding of what mental health is, the ability to identify risk and protective factors and the knowledge and attitudes needed to self-manage emotions, source support and carry out timely help-seeking [3]. Therefore, enhancing levels of MHL in school-aged students is considered a promising universal prevention strategy for mental health problems occurring in children and adolescents, and putative mechanisms include improving mental resilience, reducing stigma against mental health treatment and increasing the confidence to seek help [4–7].

The Mental Health Foundation, a registered UK charity committed to improving young people’s mental health [8], delivers a school-based, MHL programme called the Peer Education Project (PEP). Peer education, a key feature of the PEP, is a participatory style of teaching, whereby a cohort of students act as ‘peer leaders/educators’ to provide information to, and discuss sensitive issues with, a class of other students, the ‘peer recipients/learners’ [9]. Rooted in social constructivist pedagogist theories such as Social Learning Theory [10, 11], peer-relayed messages permeate social networks better than teacher-led instruction, due to peer educators who are sociologically similar to peer learners being perceived as role models [12], thus creating an environment conducive to health promotion [13, 14]. Benefits are argued to extend to peer educators too, with increased self-efficacy and self-esteem resulting from increased skill acquisition and resilience building [15]. However, they can also be exposed to risks such as feeling overwhelmed by the unfamiliarity and burden of responsibility that the role brings [16].

Systematic reviews of the effectiveness of peer-led interventions have shown promise in the areas of tobacco, alcohol and/or drug use and sexual and reproductive health [17, 18], but there is a paucity of research on mental health specifically [19, 20]. A recent review identified 11 mental health–related studies, with only two based in the UK [19]. Out of the five reporting on peer learner outcomes, two found improved self-confidence and quality of life and another found an increase in learning stress and a worsening of general mental well-being [19]. Of the seven reporting peer educator outcomes, one found improved self-esteem, one found reduced social stress and increased guilt and five found no effect [19]. The authors noted that studies lacked in exploration of mechanisms of change, impacts across different contexts and the approaches used to select, train and deliver the programmes [19]. Such process data are needed to understand why certain interventions show benefits more than others and to guide practice going forward.

An evaluation of the PEP in 2016–17 found that across six schools, peer learners’ and educators’ knowledge around mental health and confidence to discuss emotional issues improved, but generalisability was limited as the schools were mainly single sex (female only) in Southeast England [21]. In our 2020–22 evaluation across another six schools in the Southwest and North of England, we found similar improvements in mental health knowledge, help-seeking intentions and the number of sources students who were likely to seek help from, but no indication of changes in self-help, peer support or general mental well-being [22]. Exploring acceptability and potential mechanisms could help to explain why the programme improved certain aspects of MHL over others, which has not been done previously. To address this gap, we sought to conduct a realist evaluation to qualitatively explore what components of the PEP worked to produce which outcomes, how it worked and in what contexts. Our secondary aims were to identify barriers and facilitators to inform future refinements of the programme and methodological aspects that could be adapted in a randomised controlled trial to evaluate the effectiveness of the programme.

## Methods

Ethical approval for this study was obtained from the University of Lancaster’s Faculty of Health and Medicine Research Ethics Committee (reference number: FHMREC19105).

### Intervention

**Fig. 1.**
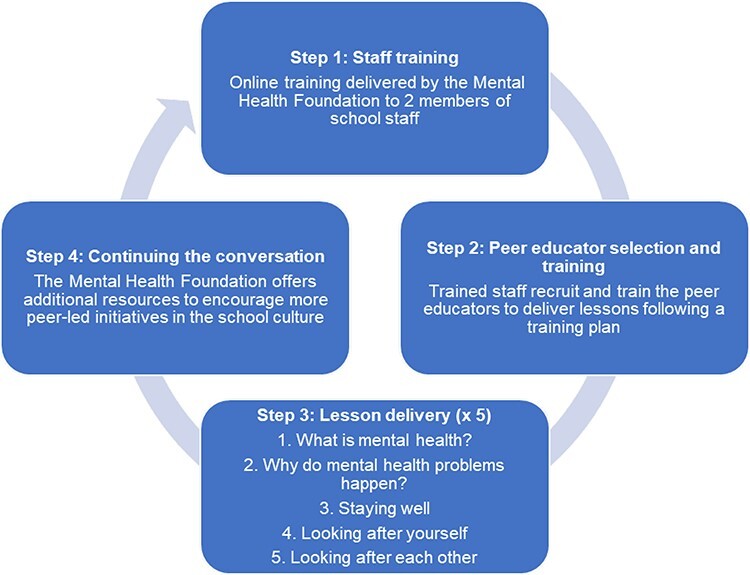
Four steps of the PEP.

The PEP consists of four steps [23] (Fig. 1). First, the Mental Health Foundation provides online training to at least two members of staff at each school to be able to deliver training to a cohort of older students. Staff training consists of a rationale behind the project, how PEP was developed, implementation and structure, access to the PEP online platform, letters for parents/guardians, guidance on peer education training and an overview of the syllabus. Second, the trained staff select ‘peer educators’ (≥14 years old/Year 10). Schools are encouraged to select 20 peer educators and consider the age and representativeness of the group, ideally from a range of backgrounds with mixed experiences of leadership roles. The staff then train the peer educators to equip them with the skills to teach the lessons following a prespecified handbook and PowerPoint slide deck and familiarise them with the principles of safeguarding. Third, the peer educators work in pairs to deliver five 50-min lessons to classes of younger students, ‘peer learners’ (≥11 years old/Year 7), at a maximum ratio of 1:20 (one peer educator per 20 peer learners). The behaviour change techniques included, which are the active components designed to change behaviour, relate to action planning, emotional and practical social support and providing information from credible source [24]. Lastly, the Mental Health Foundation seeks to continue collaborating with schools so that a whole-school approach is prioritised. This is a cyclical process, by which the staff training is continually taken up by new staff once the programme is embedded in the curriculum over time.

### Recruitment

We aimed to recruit six schools in total: four new and two current user schools, a range of school types (state schools and fee-paying schools) and free school meal status (above and below the national average for the proportion of students eligible) as a proxy for the level of catchment area deprivation [25]. As the Mental Health Foundation recommends that peer educators are 16- to 17-years-old (Year 12), schools with students up to 16 years (Year 11) were not eligible for recruitment. Schools were recruited from the Southwest and North of England for practicality reasons and to increase transferability by recruiting from two distinct geographical areas. We used a random number generator to select a total of eight schools to initially contact from our list of eligible schools (four from each area and two above/below the free school meal average). However, we only managed to recruit one school through this method as it was at the height of the COVID-19 pandemic and associated complications. Therefore, we contacted a further 46 eligible schools and recruited four on a first come, first served basis. Lastly, as we wanted to capture insights from schools already implementing the PEP, the Mental Health Foundation contacted two ‘current user’ schools directly.

### Theoretical approach

Realist evaluation reflects a distinct epistemological framework, seeking to identify the thought processes and perceptions enabled through interventions, how these vary across contexts and how these psychological mechanisms have a more profound effect on producing behaviour than the intervention inputs [26].

**Fig. 2.**
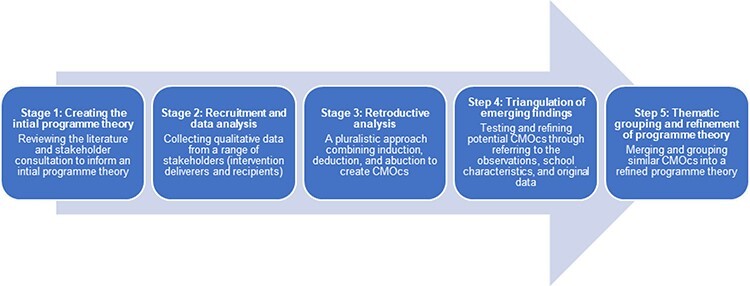
Five stages of the realist evaluation process.

**Fig. 3.**
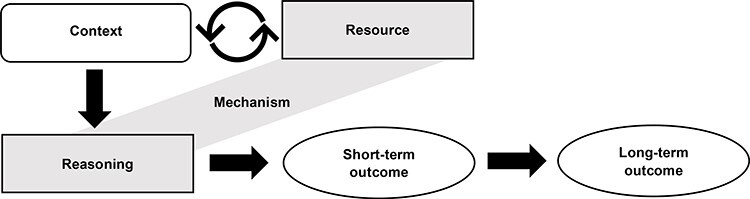
CMOc diagram.

We followed the Realist And Meta-narrative Evidence Syntheses: Evolving Standards (RAMESES II) for realist evaluations [27], and our process comprised five stages (Fig. 2). First, we developed an initial programme theory (IPT) (provided in the Supplementary Material) through conducting a systematic literature review of school-based peer education for health [20], plus an additional comprehensive literature search for other MHL programmes that were not peer-led; holding multiple consultations with our advisory group [comprising a local public health practitioner responsible for school-based Personal, Social and Health Education (PSHE) curriculum development, a school leader, a clinical academic in adolescent mental health and the lead for research at the Mental Health Foundation] and incorporating the outcomes from the aforementioned evaluation study [21]. Next, we recruited research participants and collected qualitative data to elicit views about how the PEP worked in practice. Third, we undertook a retroductive data analysis to elucidate pathways to the outcomes in the IPT and any new pathways arising from the data [28]. Retroduction uses both inductive and deductive logic as well as researchers’ insights to help identify hidden causal factors and emphasises the importance of having multiple data sources to corroborate the findings [29]. We created |Context–Mechanism–Outcome configurations (CMOcs) that separated mechanisms into ‘resources’ and ‘reasoning’ to explore how the intervention’s components (mechanism resources) activated certain responses and perceptions in the participants (mechanism reasoning) and how this interacted with the physical or social contexts in which the intervention was implemented [30]. See Fig. 3 for a graphical depiction of this feedback loop. More details on the data collection and analysis stages are provided later.

### Data collection

Data collection took the form of observations during intervention delivery, and focus groups and interviews shortly after programme completion [31]. A lead contact at each school put forward two staff members for interviews (one from the Senior Leadership Team, e.g. headteacher or deputy head, and one staff member who had been trained and/or trained the peer educators). We aimed to conduct three focus groups (two with peer learners and one with peer educators) at each school and asked staff members to recruit the students to represent a mix of genders, ethnicities and experiences. We provided letters to parents to obtain written consent for their child’s participation and took written or oral consent from the student participants prior to starting the focus groups, depending on if it was conducted in person or remotely. We provided information sheets for the staff and obtained written consent prior to the interviews commencing.

Data were collected from June 2021 to February 2022 by the main researcher (E.L.C.) and three other researchers (E.W., A.K. and M.L.). Due to the ongoing COVID-19 restrictions, we offered flexibility of online and in-person methods. The semistructured topic guides (provided in the Supplementary Material) were tailored for each stakeholder group but followed similar themes: barriers and facilitators to implementing the programme as planned; how it worked in practice; perceived impacts on students, staff, and school culture (based on the outcomes within the IPT) and desired improvements. Interviews and focus groups were recorded and transcribed verbatim, anonymised and input into NVivo (released in March 2020) for management [32]. The training and lesson observations were recorded using a structured checklist by three experienced qualitative researchers (E.L.C., E.W. and A.K.) that covered the number of attendees, delivery mode (online/in-person) and fidelity to the protocol. Example items are included in Table I.

**Table I.** School-level characteristics and data collection

	Characteristics						Data collection			
School ID	Area	User status^a^	% of free school meal eligible	School type	Peer educator year group^b^	Peer learner year group^b^	Staff interview	Peer educator focus group	Peer learner focus group	Observation
984	Southwest	New	NA^c^	Female-only boarding	12	7	2	1 (*n* = 8)	1 (*n* = 8)	2 (1 training, 1 lesson)
509	Southwest	New	14.2%	Mixed state	13	8, 9	2	1 (*n* = 10)	2 (*n* = 8, *n* = 9)	1 (lesson)
371	Southwest	Current	NA^c^	Female-only boarding	12	7, 8	1	1 (*n* = 9)	2 (*n* = 16, *n* = 21)	0
150	North	New	37.9%^d^	Mixed state	12, 13	7	3	1 (*n* = 4)	2 (*n* = 8, *n* = 4)	1 (lesson)
535	North	New	12.6%	Mixed state	13	7	1	1 (*n* = 7)	0	0
384	North	New	26.7%^d^	Mixed state	10	7	0	0	0	0
842	North	Current	12.5%	Mixed state	NA^e^	NA^e^	3	1 (*n* = 8)	2 (*n* = 6, *n* = 8)	1 (lesson)
							12	6	9	5

a New: recruited to start the intervention in 2020–21 or 2021–22; current: delivered the intervention prior to 2020–21.

b Age (years) of school year groups in the UK. Year 7: 11–12, Year 8: 12–13, Year 9 13–14, Year 10: 14–15, Year 10: 15–16, Year 12: 16–17, Year 13: 17–18.

c Data unavailable due to being independent schools, supported wholly by fee payments.

d Above national average for students eligible for free school meals at any time during the past 6 years based on 2021 data (23.7%) (GOV. UK, 2021).

e Current user school did not deliver the intervention during his study, only recruited for qualitative data collection.

### Data analysis

The main qualitative researcher (E.L.C.) developed a coding frame comprising four parent nodes(Contexts, Mechanism resources, Mechanism reasoning and Outcomes) and a priori ‘theory-driven’ codes were added as child nodes (taken directly from the constructs within the IPT). As the analysis progressed, two coders (E.L.C. and S.D.) separately coded extracts into the theory-driven codes if they supported the IPT and into new child nodes if they refuted or added to the IPT, labelled ‘data-driven’. Concurrently, we ‘linked coded’ data that directly reflected the connections between contexts,mechanisms and outcomes. Next, the two coders discussed and compared the emerging findings with the observation notes and school-level characteristics (e.g. school type and number of students) to contextualise the developing CMOcs. Lastly, we grouped the CMOcs thematically to highlight high-level mechanisms that could be generalised to similar peer-led initiatives, and collaborative discussions among the research team and practice partners were instrumental to ensuring reliability and transferability of the refined programme theory.

## Findings

### Sample

From seven schools, we conducted 12 staff interviews (*n* = 6 with Senior Leadership Team, *n* = 6 with staff trainers), 15 student focus groups (*n* = 6 with peer educators from Years 10 to 13 and *n* = 9 with peer learners from Years 7 to 9, totalling 134 students) and five observations. The following efforts were made to manage the online focus groups: a teacher was initially present to discuss expectations, and students were instructed to raise their hand on Teams/Zoom and stay on mute when not speaking to manage noise. In one school with large group numbers (ID 371), the teacher remained on the call to help facilitate the discussion. See Table II for further information on numbers and school characteristics.

**Table II.** Example topic guide, interview questions and observation field notes checklist items

Topic guide questions		Interview questions		Observation checklist items		
Peer learners	Peer educators	Staff trainers	Senior leadership team	Staff training	Peer educator training	Lesson delivery
How did you find being taught by older students?	How did you find running the sessions?	How did you find the staff training?	Were there any barriers to taking part?	Statements of understanding	Level of engagement	Use of slides and handbooks
Is there anything that was not covered?	Has taking part has any benefits for you?	How did you select peer educators?	Have you noticed any impacts so far?	Staff questions or concerns	Student questions or concerns	Issues and resolutions

### CMOcs

**Fig. 4.**
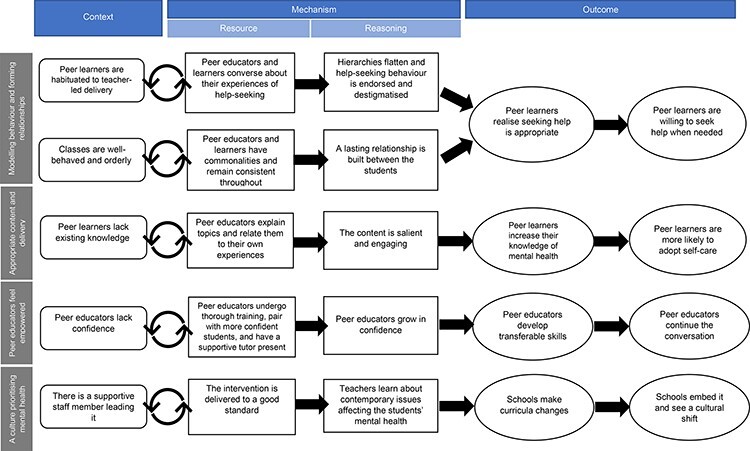
Refined programme theory comprising five CMOcs.

Our findings have been grouped into four themes: (i) modelling behaviour and forming relationships, (ii) appropriate content and mode of delivery, (iii) peer educators feeling empowered and (iV) a culture that prioritises mental health. The final five CMOcs are shown in Fig. 4, along with the theme to which they relate.

### Modelling behaviour and forming relationships

When peer learners are given the opportunity to share their feelings in a non-stigmatising environment with older students who they connect with, they recognise the importance of seeking help and feel more comfortable carrying this out in practice.

#### CMOc 1

In peer learners accustomed to traditional teacher–led lesson styles (Context), conversing with peer educators about their personal experiences of help-seeking (Mechanism resource), flattened hierarchies, endorsed, and destigmatised help-seeking behaviour (Mechanism reasoning), facilitated a realisation that seeking help was appropriate (Outcome, short-term), and a willingness to seek help when needed (Outcome, long-term).

In contrast to a more structured, didactic teaching approach, peer educators speaking about the practicalities of help-seeking, through ‘constant conversation in a formal yet informal environment’ (Senior Leadership Team, School 509), and talking ‘like we were all friends’ (Peer learner, School 509) opened the conversation up and reduced the stigma around help-seeking. Additionally, when staff were present to manage the peer learners’ behaviour, the building of supportive bonds between the peer educators and learners was facilitated and hierarchies were flattened. These informal, equitable educator-learner interactions seem to lead to peer learners feeling more at ease in disclosing their feelings overall, which, in turn, meant that they were more likely to talk about their feelings to friends and family following the programme.

Ever since the programme, I’ve talked to [my parents] a little bit more…I would say it’s good if people know things that they need to know (Peer learner, School 509).

I think it’s just gave us a bit of a better place to go – instead of just having to keep it inside, it meant that we had people to talk to and people to get anything off our chest (Peer learner, School 842).

#### CMOc 2

When lessons are well organised and orderly (Context), this ensures consistent interaction with relatable peer educators throughout the programme (Mechanism resource), which leads to lasting relationships between the students (Mechanism reasoning). This facilitates a realisation that seeking help is appropriate (Outcome, short-term) and a willingness to seek help when needed (Outcome, long-term).

The bonds formed between the students persisted beyond the lessons, especially in those who had continued contact with one set of peer educators instead of them changing partway through the intervention. This worked best in groups that were less disruptive and more engaged, which was often aided by the tutor maintaining control of the class, so that the peer educators could retain their ‘peer’ role. In turn, trusting relationships were built, and the peer learners grasped the value of seeking help and talking about their issues, which then meant that the peer educators could identify potential problems in younger students who needed following up with teachers or pastoral care staff.

The peer educators picked up that [a peer learner] was quite sad. So, with the tutor and myself, we mediated, and they’ve now built up a little relationship…led by the two students who are really keen to do it who started off saying, “we were 12 once and it’s not always easy” (Senior Leadership Team, School 509).

Participants also discussed the importance of having commonalities between educators and learners to build the desired trust and relationships throughout the intervention. This differed depending on the age and gender of the peer educators recruited. Regarding age, in Schools 984 and 150 that used the Mental Health Foundation’s recommended peer educator age group (Year 12), peer learners had mixed opinions about the appropriateness of the age gap (up to 7 years). The students from School 984 suggested that a 2-year age gap would be more favourable, so the peer educators did not ‘baby’ them as much, whereas peer learners from School 150 desired for the peer educators to be older, so they could be more ‘assertive’. This discrepancy in views could partially be explained by the observational data, as the teachers appeared more passive in the lessons in School 150 and the number of peer educators was smaller, which led to the peer educators having to take on more of a control function during the lessons and the peer educators rotating round the groups.

When it comes to talking about mental health it would have to be with someone that I trust 100% to not tell anyone…because we didn’t have that much time with [the Peer Educators] and we didn’t really see them all that much, I still don’t really trust them (Peer learner, School 150).

Regarding gender, two of the schools discussed challenges in recruiting boys as peer educators, which staff members explained may reflect ingrained issues around stigma and lack of engagement in leadership activities among males. In other schools, however, teachers felt they did not have to ‘chase anybody’ and that those who volunteered made up a diverse group.

By no means have we got the elite taking on the peer educator role…I’ve tried to keep it completely inclusive so everybody can have an opportunity…you will always get a type of student that just wants to be involved in everything, but you’ve also got some that are more late bloomers (Senior Leadership Team, School 509).

### Appropriate lesson content and mode of delivery

With the peer learners often having superficial and misleading ideas about mental health before the programme, peer educators felt a responsibility to ‘debunk’ myths portrayed on social media and highlight the adverse impacts of misinformation for mental health. However, the success of this process relied on how relevant the peer learners perceived the content to be.

#### CMOc 3

In peer learners who lacked prior knowledge of coping strategies to deal with mental health issues (Context), peer educators explaining topics and relating them to their own experiences (Mechanism resource), increased the salience of the content (Mechanism reasoning), and led to an increase in peer learners’ awareness of what mental health is, the risk and protective factors, and ways to look after their own mental health (Outcome, short-term), and an increased likelihood of adopting self-care behaviours (Outcome, long-term).

As someone who struggles with mental health, it was good to see the different coping mechanisms that I wrote down then at the time because then when I did get really bad, I could like see if they helped…so, it helped with my own mental health (Peer learner, School 150).

When peer learners were stimulated by age-appropriate content, the lessons gave them a toolbox of coping strategies to harness if their mental health became compromised. There were varied opinions among peer learners about how much new knowledge they gained, which may have been due to the slightly different ages used across schools, noting that the content was written for Year 7s. For example, Year 8s in one group felt that the content was highly relevant, whereas Year 9s in another saw it as more simplistic and repetitive.

Some stuff, like, you knew it, but when they explained it in more depth, you knew more…you go up, and up (Peer learner, Year 8, School 509).

If they went in depth about how you feel and why you feel it and quite powerful ways to stop that happening, then I think that would be a lot more useful (Peer learner, Year 9, School 509).

The content being suitable for the peer learners was discussed in depth by the teachers too, who suggested that the programme helped to foster a foundation on which to build on when revisiting the content in future years.

They need that progression…it’s obviously age-appropriate but it is a lot of information to take on board and to assimilate…things come back to them that they remember thinking, “oh yes, I remember learning about that” but if they do it over a period of time it can be built on (Staff trainer, School 371).

### Peer educators feel empowered

When peer educators felt adequately supported during the training and the lesson delivery, by both their teachers and peers, they gained confidence and a wealth of practical, social and emotional skills.

#### CMOc 4

In peer educators with low confidence (Context), undergoing thorough training, pairing with more confident peer educators and having a supportive tutor present (Mechanism resource) enabled them to grow in confidence (Mechanism reasoning), develop organisational, presentational and leadership skills (Outcome, short-term), and continue the conversation with the peer learners (Outcome, long-term).

The training the peer educators received was generally perceived in a positive light, with peer educators commending the structure and the chance to collaborate and share ideas with others. However, suggestions were made regarding the timing and delivery, e.g. a shorter time gap between the training and lesson delivery, having ‘a supervised lesson like at the beginning with like help from a teacher’ (Peer educator, School 509) or undergoing refresher training from the Mental Health Foundation part way through the programme ‘rather than it just being school-led’ (Staff trainer, School 984). In terms of the content of the training, peer educators desired more advice on using the resources as prompts to avoid reading them word-for-word and dealing with emotional problems they might face as a result of taking part.

During the lesson delivery, those who were less experienced relied more heavily on the teachers to deal with immediate issues ‘if someone was being a bit naughty or there were a couple of kids who were a bit upset’ (Peer educator, School 535) or their partners who were perhaps more ‘used to volunteering’ (Staff trainer, School 150), but their confidence gradually grew. Specific examples included the programme spurring one student to aspire ‘to be a teacher’ (Staff trainer, School 150) and another to boost her academic potential, ‘who struggled to get into sixth form because of her confidence and her levels in her language skills, yet has just bloomed’ (Senior Leadership Team, School 509). This development of confidence naturally led to building social and transferable skills, including ‘how to work in a team’ and ‘how to get things done efficiently’ (Peer educator, School 842), which led to peer educators feeling comfortable with the idea of supporting younger students in the future.

We’re aiming to set up the sort of thing where young students can come and talk to the older students and student parliament…because we’ve gone through this course, we kind of have an idea of how to deal with the topic…even if it’s like a Year 10 (Peer educator, School 509).

In some cases, with the programme enhancing the peer educators’ skills, consolidating their knowledge on how to protect their own mental health and allowing them to share their personal stories, this led to them feeling that their well-being had improved as an additional long-term outcome.

It also helped one of the girls…a recovering anorexic so just giving her a voice and being able to give her a sense of purpose is very powerful…delivering something on the benefits of good mental health, can only do good I would have thought for those who deliver it (Staff trainer, School 150).

### A culture prioritising mental health

Having ‘the right person driving it…who knows what the students need’ (Senior Leadership Team, School 984) from the outset was perceived as a key contextual prerequisite to reinforce the programme’s benefits, both in the short term, during delivery, and long term, following its completion. Continued support from this staff member ensured fidelity to the intervention protocol, e.g. through identifying and overcoming barriers around timetabling and recruitment, ensuring that the peer educators represented the general student body and enabling the staff to feel adequately trained themselves before training the peer educators.

**CMOc 5:** In schools with an invested staff member (Context), the intervention is implemented to a good standard (Mechanism resource), leading to the teachers themselves learning about contemporary issues (Mechanism reasoning), schools making curricula changes, increasing access to help sources (Outcome, short-term) and embedding the programme to drive a cultural shift (Outcome, long-term).

I would absolutely endorse it [continuing the programme for next year]. I very much leave that to my head of PHSE because she’s doing all the hard work and I’m just the one going yes, that’s brilliant (Senior Leadership Team, School 984).

Staff training was experienced variably across schools, but the participants alluded to benefits of both in-person and online delivery, with in-person sessions offering more opportunity for ‘dynamic discussion’ (Staff trainer, School 371), while the online webinars were more focused and flexible. When asking staff whether they felt prepared to train peer educators following the training, they often reflected on their existing role within the school, with those who were more experienced teachers feeling more confident, while the teaching assistants desired ‘some sort of video or visual demonstration as to how to teach’ (Staff trainer, School 150) or more support from higher up to enable teacher involvement. By contrast, a staff trainer in a school that had been implementing the programme for several years noted that teacher involvement required a level of acceptance that the programme would require a significant, albeit worthwhile, time commitment.

Doing training in the evenings after school…was brilliant, because you could have your camera off, you could watch and just write loads of notes (Staff trainer, School 984).

It’s a body of work…it took me out of the classroom for three weeks which is not exactly a barrier, it’s just one of those things where if we want to run it then that’s got to happen (Staff trainer, School 842).

Following the programme, teachers reflected on what they learnt about novel issues and sources of support through the training and lessons. These insights often then informed cultural changes, e.g. additions to the PSHE curriculum, including new online help sources, forging stronger links between younger students and the student parliament in the older years, plans to embed the programme, so it is delivered throughout the academic year and providing further formal training for staff as they became more alert to signs of poor mental health among students.

Interesting a lot of the signposting was to websites, which obviously we wouldn’t have had when I was younger…we do things about social media in PSHE a lot because obviously that’s how a lot of people are running their social life now…we also opened a new section on the website…there’s about 20 links in there for all different sorts of things (Staff trainer, School 371).

We treat it like a safeguarding issue, so they’ll use our safeguarding protocols as a way of raising concerns which is absolutely fine, so that’s how a lot of those come in (Senior Leadership Team, School 842).

## Discussion

To the author’s knowledge, this is the first realist evaluation in the field of adolescent mental health, and we feel that our study provides a strong example of how to apply this methodology to a school-based intervention. We aimed to identify the mechanisms of change and how these were impacted by the context in which it was implemented. Mechanisms clustered into four overarching themes, relating to peer educators modelling behaviour and building peer-to-peer relationships, the peer learners perceiving the content and delivery mode as appropriate, peer educators feeling empowered and the programme engendering a school culture that prioritises mental health support. Our findings support previous research around the pathways through which peer education improves knowledge, attitude and behaviour and provides novel insights into mental health specifically.

Concerning peer learner outcomes, our findings suggest that role modelling and relationship building worked to increase willingness to discuss feelings and seek help when needed. As perceived commonality was an important determinant of this, future programmes could consult peer learners to find an age group that enables optimal ‘social distance’ for both similarity and authority and select peer educators from certain demographic backgrounds such as gender, ethnicity and age to enhance identification, empathy and sustainability of cross-year group communication [33]. This could increase students’ ‘affiliation’ to the school, a key pillar of a health-promoting environment [34, 35]. While in our quantitative analysis [22], the effects did not differ between the schools that selected non-Year 12s as peer educators, or non-Year 7s as peer learners, this moderating effect could be explored more robustly in a larger study.

Our second theme describes how peer educators delivering age-appropriate content, and relating it to their own experiences, increased the salience and acceptance of messages. Similarly, previous research has suggested that relational conversation, rather than didactic instruction, and collaborative rather unanimous decisions on the content of the sessions can promote longer-lasting peer-led intervention effects [36, 37]. This suggests that the lesson content could be co-developed in the future to ensure that issues of most relevance to peer learners are incorporated.

Our third theme highlights a connection between enhancing the peer educators’ skills and confidence, their sense of self-worth and their willingness to comfort others in future interactions [33]. The peer educators in our sample varied in their baseline experience, and less experienced students relied more heavily on the lesson plans and teachers’ support, e.g. to maintain order in the classroom. This suggests the importance of providing adequate supervision and targeted training to ensure that peer educators are comfortable in facilitating a large group. Given that young people who are vulnerable to mental health problems, e.g. by virtue of belonging to a marginalised group [38], may also be less confident in their abilities to teach in this way, these insights suggest that training vulnerable students, rather than groups of ‘high achievers’ or regular volunteers, could be a way to reduce mental health inequalities [39, 40]. Directing and supporting diverse sets of peer educators, allowing them to adapt as they go, could promote an ethos of teamwork and ‘practical reasoning’ to appreciate and reflect on the breadth of perspectives people hold, which is another central tenet of health-promoting schools [34].

The fourth theme is related to how the programme engendered a shift in ‘school culture’, defined as the shared beliefs and norms across the population that influence psychological and physical well-being [41–43]. We found that the PEP promoted an increased awareness of existing sources of support amongst students and staff and often led to the staff making tangible changes to the support infrastructure, aligning with the whole-school approach that has been identified as a governmental priority [44]. Through this cultural shift, stigma around speaking about mental health dissipated and destigmatisation is a factor known to protect adolescent mental well-being [5]. Aligning with previous evaluations and the Diffusion of Innovations Theory [45, 46], whereby effective implementation relies on ‘early adopters’ being influential among the social network [45, 47, 48], the cultural shift was precipitated by an invested staff member reflexively supporting and refining the intervention.

### Implications

Our refined programme theory provides suggestions on how to improve the PEP to maximise effectiveness, all of which could be applied to the quality of peer-led interventions in other health areas. The staff training being online was acceptable, but the content could have been improved by more practical advice given to teaching assistants with less teaching experience. Our insights also suggested that providing ongoing training partly delivered by the charity partner and taking a targeted approach to support less experienced students could increase peer educators’ confidence in their abilities. Third, we suggest that schools align with the recommended five lessons being delivered over a single term and use the target peer learner age group (Year 7/8) as the content is specifically tailored to meet their knowledge requirements. The optimal age of the peer educators is less clear, but our findings suggest that a slightly younger target group such as Year 10/11 could promote more empathy and rapport, while also increasing the reach of the intervention into schools with year groups up to age 16 years. During the lesson delivery, the plans should allow time for informal discussion alongside the structured content, and the teacher’s role could be better clarified. This is important as we found that the presence of the class tutor did not dilute but, in fact, enhanced the peer-to-peer dynamic. Relatedly, we highlight the significance of having a key staff member with the capacity, experience and motivation to support the organisation and timetabling of the programme, to enhance its integration into the school culture.

### Strengths and limitations

There are several strengths and limitations of our study. Collecting data from a range of school types and sources, combining stakeholder interviews, focus groups and observations, enabled rich and detailed data about the processes and contexts that are needed for effective implementation and the selection of illustrative quotes that reflected sentiments across the whole sample. Regular team discussions ensured a reflexive retroductive analytic process that considered the existing theory, empirical data and emergent theory to identify the most plausible and transferable explanations underpinning the observed outcomes [5]. Furthermore, as most studies focus on constructs around mental ill health, rather than knowledge of prevention and wider health promotion [3], we feel as though our study provides an exemplar for how to qualitatively evaluate impacts on preventative aspects such as help-seeking, self-care and broader promotion of well-being.

Regarding limitations, COVID-19 posed challenges for recruitment, and we struggled to recruit as many schools as we planned in more deprived catchment areas, thus making it difficult to ascertain the impact of the intervention on mental health inequalities. We did not collect demographic data from individual participants, so the representativeness of the sample remains unclear. Also, it is unclear whether the intervention had certain effects due to the loneliness and restricted access to support during the lockdown periods. A further limitation is that while we attempted to adhere to the realist approach in its entirety, our IPT did not include prespecified CMOcs [30], and therefore, our programme theory warrants testing in a larger-scale study. This would also aid with understanding the PEP’s impact on MHL outcomes that have only been evaluated quantitatively in small-scale, short-term studies [21, 22] and are variables that are inherently hard to measure, with MHL being a multifaceted, contested construct [3].

## Conclusion

Our realist evaluation not only provides a unique contribution to the small evidence base on peer-led school-based MHL interventions but has also generated findings of relevance to MHL education and peer-led health interventions more generally. In line with the IPT, we found that peer learners increased their knowledge and intentions around seeking help and self-care, peer educators enhanced their social skills and staff made curricula changes and plans to embed the programme. Novel insights included the importance of teachers maintaining order in the classroom, so the peer educators could flourish, the selection of relatable peer educators being integral to building trusting relationships and a key staff member driving concerted effort across the whole school. Our refined programme theory could be used to inform improvements to the PEP and other peer-led programmes in schools. Beyond teaching MHL, our findings also underline the importance of schools embedding a sense of open discussion of mental health and well-being and continuing developing school cultures that are supportive of and prioritise good mental health.

## Supplementary Material

cyad026_Supp
