# Supplementary material for: Exploring mechanisms and contexts in a Peer Education Project to improve mental health literacy in schools in England: a qualitative realist evaluation
Source: Health Educ Res. 2023 Jul 24;39(1):40–54. doi: 10.1093/her/cyad026 (PMC10805377; doi:10.1093/her/cyad026)
Supplement: cyad026_Supp [file cyad026_supp.zip › Supplementary material.doc]

Systems of mental health support that are clear/known to pupils

A school culture that promotes mental health literacy and is open to discussion of challenges to mental health

Senior leadership team who are supportive of the intervention

Improved and sustained mental health literacy

Increased likelihood of effective self-help behaviours

Increased likelihood of help-seeking for mental health problems

Supportive of each other’s mental health

Continued engagement with learning about mental health

Improved organisational, presentational and leadership skills

**Context**

Training of at least 2 school staff

Delivery of mental health literacy sessions to years 7 or 8 (11-13 years)

**Mechanisms of change**

Peer educators are more credible sources of information

Peer educators have greater empathy for and understanding of the challenges faced by learners

Peer educators adopt less didactic approaches to imparting information, leading to a flattening of the hierarchies normally present in teacher-student relationships

Peer educators model appropriate behaviours and endorse key messages

**Outcomes**

**Short term**

**Long term**

Peer educators and peer learners

Project embedded in curriculum year on year

Prioritisation of further in-touch points with pupils around mental health

**School**

**Intervention**

**School level**

Visible school commitment to importance of mental health creates school culture that is more open to discussion and support of mental health

Intervention is universal, so creates cultural change across whole year group of learners

A cohort of confident, informed Peer educators share their knowledge and insights formally and informally throughout the school

**Classroom level**

**Peer educators**

Peer educators and peer learners

Increased understanding of what mental health is, and awareness of risk and protective factors

Increased knowledge of ways to look after own mental health

Increased awareness of sources of support

Increased understanding of how to support others

Peer educator selection (aged 14+), training and ongoing support

**Year 7 Focus Group Topic Guide**

**Study Title: Mental Health Peer Education Project**

**Icebreaker Session:** Students to create a ‘graffiti wall’ by writing down everything they can remember learning from the Peer Education Project.

1. How did you find the Peer Education Project?
2. Was it what you expected?
3. How did you find being taught by older pupils?
   - Do you prefer being taught by teachers?
   - Why/why not?
   - What do you think was different about having peer educators instead of teachers? (More boys?)
4. What were your favourite parts of the programme?
5. What were your least favourite parts of the programme?
6. Is there anything you would change about the programme/lessons?
7. Did you feel the older pupils represented your school well?
8. Has the course made you more likely to speak with sixth formers in the future or ask them for help?
9. Has the programme impacted your views/behaviour around mental health?
   - How? What did you learn?
10. Has it had any other benefits for you?
    - Confidence, self-esteem, social skills, increased knowledge etc.
11. Any other benefits for your peers?
12. Any changes in the way your classmates behave towards each other?
13. Any changes to how you behave towards your friends?
14. What worked well?
15. What could work better?
16. Is there anything you would like to learn about that wasn’t covered?
17. Have you changed anything you do/don’t do following lessons?
18. What else does your school do to support student mental health?
    - Is there anything you’d like your school to do?
19. Finally how did you find the survey you had to fill out? Did you do it at home or during a lesson?
20. And how did you find the focus group today?

- Can anyone think of any good things about talking about the project like we did today? Any benefits for you?

Is there anything else you would like to add that we haven’t covered?

**Peer Educator Topic Guide**

**Study Title: Mental Health Peer Education Project**

**Training/Selection**

1. Why did you want to get involved in this project?
2. Was it easy to get involved? Any barriers?
3. What aspects of the training did you enjoy/not enjoy?
4. Did you feel ready to teach younger students after the training?
5. Would you have changed anything about the training?
6. What was harder / easier than you expected?
7. Did you have ongoing training and support from the teachers throughout the sessions?

**Delivery**

1. How did you find teaching younger pupils in your school?
2. Were there any sessions you particularly liked teaching?
3. Was it what you expected?
4. What were your favourite parts of the programme?
5. What were your least favourite parts of the programme?
6. Is there anything you would change about the programme/lessons?
   - What works well and what could work better?
   - What helped it go well / what would have helped it go better?

**Impact on Peer Educators**

1. Did the peer education project impact on your school work?
2. Has the programme impacted your views/behaviour around mental health?
   - If so, how?
3. How did you find working with the other peer educators?
   - Did you feel there was a good mix of peer educators?
4. Has it had any other benefits for you? Personal impact?
   - Confidence, self-esteem, social skills, increased knowledge etc.
5. Any other benefits for your peers?
6. Would you recommend a friend did this?
7. What else does your school do to support student mental health?
   - Is there anything you’d like your school to do?

Is there anything else you would like to add that we have not covered?

**Senior Leadership/Pastoral Care Interview Topic Guide**

**Study Title: Mental Health Peer Education Project**

1. How did you hear about the Peer Education Project?

- Was it easy to get involved?
- Were there any barriers to getting involved?

2. If applicable (existing user schools)

- How many years have you been delivering the PEP?
- Has the programme changed at all since you started delivering it?
  - How did you implement it this year compared to other years?
- How well is the PEP embedded in your curriculum? (if PEP already implemented)

3. What are your views on the Peer Education Project?

- Likes/dislikes?
- Any parts of the programme you would change?
- Views on peer educators delivering to Year 7s?

4. How does PEP compare to any other interventions to support student mental health and wellbeing in your school?

- Similarities/differences

5. Have you noticed any impacts so far?

- Attitudes/behaviour of pupils?
- Wider impact on students, staff, the school as a whole?

6. Any suggestions on how best to train teachers in the future?

- Full day (face-to-face)?
- Series of webinar training sessions?

7. Any suggestions on how best to recruit peer educators to ensure a diverse group of students?

- How representative did you feel the students were of the school’s context and overall?

8. What advice would you give other schools taking part?

- Lessons learned
- Would you recommend to other schools?

9. Lastly just wanted to touch on your school’s involvement in the study in general. So how did you find administering the survey?

- I gather that you *couldn’t allocate lesson time*?
- Do you have a gist of how many pupils were absent at that time?
- If possible/not already completed - fill out Google form together: [https://forms.gle/qr1e5QizNkvgyDXP6](https://eur02.safelinks.protection.outlook.com/?url=https%3A%2F%2Fforms.gle%2Fqr1e5QizNkvgyDXP6&data=04|01|doddsr2@live.lancs.ac.uk|a85e176aeb0f44291a6b08d99219adf4|9c9bcd11977a4e9ca9a0bc734090164a|0|0|637701461253404838|Unknown|TWFpbGZsb3d8eyJWIjoiMC4wLjAwMDAiLCJQIjoiV2luMzIiLCJBTiI6Ik1haWwiLCJXVCI6Mn0%3D|1000&sdata=1wK5tyEdBYUJ4Hii8AeEZM2foqWhdoiEkL%2BolCTbEtY%3D&reserved=0)
- And what about the focus groups and interviews? Do you think the students enjoyed this aspect?

Is there anything else you’d like to add that we haven’t covered?

**Staff Receiving/Delivering Training Interview Topic Guide**

**Study Title: Mental Health Peer Education Project**

1. How did you find the PEP training?

- Is it what you expected?
- Length/format/depth
- Why were they chosen to do this?

2. After the training did you feel equipped to train the student peer educators?

- Any further support required?
- Any barriers?

3. How did you approach recruiting students to be peer educators?

- Barriers?
- Enablers?
- How representative did you feel they were of the school as a whole?

4. How do you feel about the programme approach?

- Would you change anything?
- What worked well and what could work better? Any gaps?

5. What were the barriers/facilitators to your school taking part?
6. Has the intervention had any impact so far?

- Attitudes/behaviour of pupils?
- Wider impact on staff / school / community
- Did they gain anything regarding their own MH from the training?

7. Any suggestions on how best to train teachers in the future?

- Full day (face-to-face)?
- Series of webinar training sessions?

8. What was the process of selecting peer educators?

- Do you feel the selected peer educators represented the school well?
- Did you have/how did you ensure a diverse group of peer educators?

9. What advice would you give other schools taking part?

- Lessons learned
- Would you recommend to other schools?

10. Lastly just wanted to touch on your school’s involvement in the study in general. So how did you find administering the survey?

- I gather that you *couldn’t allocate lesson time*?
- Do you have a gist of how many pupils were absent at that time?
- If possible/not already completed - fill out Google form together: [https://forms.gle/qr1e5QizNkvgyDXP6](https://eur02.safelinks.protection.outlook.com/?url=https%3A%2F%2Fforms.gle%2Fqr1e5QizNkvgyDXP6&data=04|01|doddsr2@live.lancs.ac.uk|a85e176aeb0f44291a6b08d99219adf4|9c9bcd11977a4e9ca9a0bc734090164a|0|0|637701461253404838|Unknown|TWFpbGZsb3d8eyJWIjoiMC4wLjAwMDAiLCJQIjoiV2luMzIiLCJBTiI6Ik1haWwiLCJXVCI6Mn0%3D|1000&sdata=1wK5tyEdBYUJ4Hii8AeEZM2foqWhdoiEkL%2BolCTbEtY%3D&reserved=0)

11. If applicable (existing user schools)

- How many years have you been delivering the PEP?
- Has the programme changed at all since you started delivering it?
- How well is the PEP embedded in your curriculum?

Is there anything else you’d like to add that we haven’t covered?
